# Supplementary material for: The introduction of an invasive weed was not followed by the introduction of ethnobotanical knowledge: a review on the ethnobotany of Centaurea solstitialis L. (Asteraceae)
Source: PeerJ. 2023 Jun 7;11:e15489. doi: 10.7717/peerj.15489 (PMC10257394; doi:10.7717/peerj.15489)
Supplement: Supplemental Information 1 [file peerj-11-15489-s001.docx]

Supplemental Table 1. Traditional uses of *C. solstitialis* organized by country, common name, category of use, part of the plant used, specific uses, preparation and the author of the respective study

| **Country** | **Region** | **Common name** | **Category of use** | **Part used** | **Specific uses** | **Preparation** | **References** |
| --- | --- | --- | --- | --- | --- | --- | --- |
| Iran | Alvand and Tuyserkan | ʧæᴕertekʌnґ (in phonetics) | Medicinal | whole plant | Treatment of kidney stones | Decoction | Mosaddegh et al. (2015) |
| Iran | Hamedan | ʧæᴕertekʌnґ (in phonetics) | Medicinal | whole plant | Treatment of kidney stones | Decoction | Naghibi et al. (2014) |
| Iran | North Khorasan province, Farouj district | Gole gandome zard | Edible | flowers; roots | Food | not specified | Farouji & Khodayari (2016) |
| Iran | North Khorasan province, Farouj district | Gole gandome zard | Medicinal | flowers; roots | Refrigerant, Stomach tonic | not specified | Farouji & Khodayari (2016) |
| Iran | Shiraz | no information | Medicinal | no information | antipiretic | no information | Adeghi & Borjian (2013) |
| Iran | South of Kerman | Golgandom zard | Medicinal | aerial parts | Eye diseases, skin rash | Liniment | Sadat-Hosseini et al. (2017) |
| Iran | Urmia | Gol Gandome Zard | Medicinal | Inflorescence | treatment of dysentery | Decoction | Bahmani et al. (2014) |
| Iran | West Azerbaijan, Ghasemloo Valley | no information | Medicinal | no information | not specified | no information | Mohammadi & Azar (2012) |
| Italy | Latium | no information | Edible | Shoots | preparation of "Acquacotta", a soup made of wild plants | Cooked | Guarrera (1994) |
| Italy | Sicily | no information | Edible | no information | food | Boiled | Lentini & Venza (2007) |
| Italy | Sicily | aprocchio, apuarchiu, procchia | Edible | leaves, whole plant | Food | Boiled vegetables, fried as vegetables with eggs | Licata et al. (2016) |
| Italy | Sicily | Apròcchiu fimminedda, Gattaredda | Edible | Leaves | food | Cooked | Geraci et al. (2018) |
| Italy | Sicily, Madonie Regional Park | Gattaredda | Medicinal | whole plant | diuretic | Condiment | Leto et al. (2013) |
| Jordan | Al-Mafraq | no information | Medicinal | aerial parts | treatment of kidney stones | Fresh, soaked, cool and oral taken one time a day | Al-Quran (2015) |
| Pakistan | Northwest | Barham dandi | Medicinal | whole plant | febrifuge | 2 spoons of powdered form are taken orally with water 3 times a day for 3 days | Adnan et al. (2014) |
| Saudi Arabia | Sarrwat Mountains at Taif | no information | Edible | no information | not specified | no information | Al-Sodany et al. (2013) |
| Saudi Arabia | Sarrwat Mountains at Taif | no information | Medicinal | no information | not specified | no information | Al-Sodany et al. (2013) |
| Turkey | Yalova (northwest) | Çakırdikeni | Medicinal | Flowers | treatment of chill | dried flowers are taken internally every morning | Koçyi̇ği̇t & Özhatay (2006) |
| Turkey | Adana | Çakir Dikeni | Medicinal | Stem | woman’s/man’s diseases, tonic, respiratory disorders, mouth sore (human and animal) | Fresh | Akbulut (2015) |
| Turkey | Anatolia region (Afyon Karahisar) | Güllüdiken | Medicinal | Flowers | treatment of common colds | infusion, tea | Honda et al. (1996) |
| Turkey | Çanakkale, Bayramiç | Gelindili, Çakır diken, Çakırca, Çakırca diken | Medicinal | aerial parts | treatment of malaria | Decoction | Bulut & Tuzlaci (2015) |
| Turkey | Denizli | Korugoz, Sarı diken | Medicinal | Spine of involucral bracts | treatment of boil (furuncle) | Crushed | Bulut et al. (2017) |
| Turkey | Denizli | Korugoz, Sarı diken | Medicinal | aerial parts | treatment of common cold in animals | boiled and vapors inhaled by animals | Bulut et al. (2017) |
| Turkey | East Anatolia | Belhok | Medicinal | green aerial parts | treatment of malaria | Decoction | Altundag & Ozturk (2011) |
| Turkey | Edirne, Lalapaüa | Çakır dikeni, Kavgalaz dikeni, Kaynanadili | Medicinal | Capitula | treatment of malaria | Crushed and taken orally | Tuzlacı et al. (2010) |
| Turkey | Edirne, Uzunköprü | Sarı tiken | Medicinal | Spines | treatment of warts | Spines are pricked in wart | Güneş (2017) |
| Turkey | Inner Taurus Mountains | Oglak dikeni | Medicinal | Flowers | treatment of malaria | taken orally like pills or smoked like a cigarette | Yesilada et al. (1995) |
| Turkey | Inner Taurus Mountains | Oglak dikeni | Medicinal | flowers and leaves | treatment of stomach upset and abdominal pain | flowers taken orally while soft (rolled up into pills) or decoction of dried leaves to drink | Yesilada et al. (1995) |
| Turkey | Karaköy village, Vezirköprü-Samsun | Çakırdikeni | Medicinal | Seeds | treatment of herpes | roasted and powdered then applied to affected zone | Fujita et al. (1995) |
| Turkey | Manisa, Turgutlu | Çakirdiken | Medicinal | Capitula | treatment of malaria | Infusion | Bulut & Tuzlaci (2013) |
| Turkey | Middle Aegean Region | Süpürge out | Material culture | stems and branches | The aerial parts are cut and made into a bundle and tied into a broom used to sweep threshing area and streets | The aerial parts are cut and made into a bundle and tied into a broom used to sweep threshing area and streets | Kargıoğlu et al. (2010) |
| Turkey | Muğla | çakırdikeni/kababafldikeni | Edible | Leaves and stem | food; medicinal and veterinary (not specified) | The spines and green parts of the leaves are removed and only the large central veins are left. These are boiled, and a filling of rice, onions and spices is placed in the middle of the veins, which are then folded over and tied with string. After they are cooked in a pan, they are dipped in a mixture of flour, eggs and water, and fried in hot olive oil | Ertug (2004) |
| Turkey | South-East Anatolia, Şanlıurfa | Çakırdikeni | Edible | aerial parts | food/pastry | not specified | Akan et al. (2013) |
| Turkey | West Anatolia | Çakır dikeni | Edible | aerial parts | food for sheep during winter | Dry | Kargıoğlu et al. (2008) |
