# Supplementary material for: The introduction of an invasive weed was not followed by the introduction of ethnobotanical knowledge: a review on the ethnobotany of Centaurea solstitialis L. (Asteraceae)
Source: PeerJ. 2023 Jun 7;11:e15489. doi: 10.7717/peerj.15489 (PMC10257394; doi:10.7717/peerj.15489)
Supplement: Supplemental Information 2 [file peerj-11-15489-s002.docx]

Supplemental Table 2. List of volatile compounds identified in *C. solstitialis* plants, organized by type of compound (terpene or nonterpene), relative abundance range, region of origin, parts of the plant analysed and the authors of the respective study

|  | **Compounds** | **Treshold (%)** | **Regions** | **Parts of the plant** | **References** |
| --- | --- | --- | --- | --- | --- |
| **Terpene compounds** | |  |  |  |  |
|  | (E)-α-bergamotene | 0.07 - 0.2 | Turkey | Flower buds; Flower heads | Binder et. al. (1990) |
|  | (E)-β-Farnesene | 0.13 - 0.67 | California; Italy; Turkey | Flower buds; Flower heads | Senatore et. al. (2015); Buttery et. al. (1986); Binder et. al. (1990) |
|  | (E)-β-ionone | 1.1 | Iran | Aerial parts | Esmaeili et. al. (2006) |
|  | (Z)-Nerolidol | 0.2 | Italy | Flower heads | Senatore et. al. (2015) |
|  | (Z)-α-Santalol | 0.2 | Italy | Flower heads | Senatore et. al. (2015) |
|  | 1,8-cineole | 9.6 | Iran | Aerial parts | Esmaeili et. al. (2006) |
|  | Acroptilin | 0.01 - 5.75 | Argentina; Australia; California; Chile; Spain; Turkey | Leaves | Irimia et. al., 2018 |
|  | Aguerin B | tr - 0.54 | California; Chile; Spain; Turkey | Leaves | Irimia et. al., 2018; Sotes et. al. (2015) |
|  | Aromadendrene | 0.3 | California; Italy | Flower heads | Senatore et. al. (2015) |
|  | Bicycloelemene | 0.4 | Croatia | Aerial parts | Carev et. al. (2017) |
|  | Bicyclogermacrene | 0.32 - 7.2 | California; Croatia; Turkey | Aerial parts; Flower buds; Flower heads; Leaves/stems | Carev et. al. (2017); Buttery et. al. (1986); Binder et. al. (1990); Oster et. al. (2015); Beck et. al. (2013) |
|  | Calamenene | 0.04 | Turkey | Flower buds | Binder et. al. (1990) |
|  | Calarene | 0.5 | Croatia | Aerial parts | Carev et. al. (2017) |
|  | Caryophyllene | 0.59 - 4.3 | California; Italy; Turkey | Flower buds; Flower heads; Leaves/stems | Senatore et. al. (2015); Buttery et. al. (1986); Binder et. al. (1990) |
|  | Caryophyllene oxide | 1.5 - 25.2 | Algeria; Iran ; Italy | Aerial parts; Flower heads | Esmaeili et. al. (2006); Senatore et. al. (2015); Lograda et. al. (2013) |
|  | Cebellin F | 0.1 - 0.22 | Australia; California; Spain; Turkey | Leaves | Irimia et. al., 2018 |
|  | Cyclosativene | 0.1 | Italy | Flower heads | Senatore et. al. (2015) |
|  | Cyclosativene | 0.59 - 2.11 | Turkey | Flower buds; Flower heads | Binder et. al. (1990) |
|  | Cynaropicrin 3,4´diacetate | 0.1 - 0.26 | Argentina, California; Spain, | Leaves | Irimia et. al., 2018 |
|  | Cynaropicrin 3-acetate | 2.97 - 8.83 | Argentina; Australia; California; Chile | Leaves | Irimia et. al., 2018 |
|  | Cynaropicrin 4´-acetate | 0.16 | California | Leaves | Irimia et. al., 2018 |
|  | Cyperene | 0.3 | Italy | Flower heads | Senatore et. al. (2015) |
|  | Desacylcynaropicrin | 0.32 - 1.92 | Argentina; California; Chile; Spain; Turkey | Leaves | Irimia et. al., 2018; Sotes et. al. (2015) |
|  | E,E-α-Farnesene | - | California | Leaves | Beck et. al. (2013) |
|  | Elemol | 2.7 | Algeria | - | Lograda et. al. (2013) |
|  | Epi-cubenol-1 | 1.37 | Algeria | - | Lograda et. al. (2013) |
|  | Epoxyrepdiolide | 0.77 - 19.07 | Argentina; Australia; California; Chile; Spain; Turkey | Leaves | Irimia et. al., 2018; Sotes et. al. (2015) |
|  | Eudesmol | 2.51 | Algeria | - | Lograda et. al. (2013) |
|  | Germacrene D | 1.65 - 15.3 | Algeria; California; Croatia;  Iran;  Italy;  Turkey | Aerial parts; Flower buds; Flower heads; Leaves/stems | Esmaeili et. al. (2006); Carev et. al. (2017); Senatore et. al. (2015); Buttery et. al. (1986); Binder et. al. (1990); Oster et. al. (2015); Beck et. al. (2013); Lograda et. al. (2013) |
|  | Humulene | 0.1 - 0.3 | Turkey | Flower buds; Flower heads; Leaves/stems | Binder et. al. (1990) |
|  | Humulene epoxide II | 0.2 - 1.1 | Iran ; Italy | Aerial parts; Flower heads | Esmaeili et. al. (2006) |
|  | Janerin | 0.77 - 19.85 | Argentina; Australia; California; Chile; Spain; Turkey | Leaves | Irimia et. al., 2018; Sotes et. al. (2015) |
|  | Limonene | 0.05 - 3.3 | Algeria; California;  Iran;  Turkey | Aerial parts; Flower buds | Esmaeili et. al. (2006); Buttery et. al. (1986); Binder et. al. (1990); Lograda et. al. (2013) |
|  | Linalool | tr - 0.5 | Iran; Italy | Aerial parts; Flower heads | Esmaeili et. al. (2006); Senatore et. al. (2015) |
|  | Linichlorin A | tr - 9.62 | California; Chile; Spain; Turkey | Leaves | Sotes et. al. (2015) |
|  | Longifolene | 3.6 | Croatia | Aerial parts | Carev et. al. (2017) |
|  | Lupeol | tr - 0.22 | California; Chile; Spain; Turkey | Leaves | Sotes et. al. (2015) |
|  | Lupeol acetate | tr - 0.15 | California; Chile; Spain; Turkey | Leaves | Sotes et. al. (2015) |
|  | Myrcene | 0.02 - 0.7 | California;  Iran;  Turkey | Aerial parts; Flower buds | Esmaeili et. al. (2006); Buttery et. al. (1986); Binder et. al. (1990) |
|  | *p*-cymene | 0.1 - 1.3 | California;  Iran | Aerial parts; Flower buds | Esmaeili et. al. (2006); Buttery et. al. (1986) |
|  | Repin | 9.31 - 42.4 | Argentina; Australia; California; Chile; Spain; Turkey | Leaves | Irimia; Sotes et. al. (2015) |
|  | Sabinene | 0.3-3.26 | Algeria; Iran | Aerial parts | Esmaeili et. al. (2006); Lograda et. al. (2013) |
|  | Solstitialin | 0.08 - 1.44 | California; Chile; Spain; Turkey | Leaves | Sotes et. al. (2015) |
|  | Solstitialin A-13 acetate | 0.85 - 10.3 | Argentina; Australia; California; Chile; Spain; Turkey | Leaves | Irimia et. al., 2018; Sotes et. al. (2015) |
|  | Solstitialin A-3 acetate | tr - 0.94 | California; Chile; Spain; Turkey | Leaves | Irimia et. al., 2018; Sotes et. al. (2015) |
|  | Solstitialin A-3, 13 diacetate | tr - 8.1 | California; Chile; Spain; Turkey | Leaves | Sotes et. al. (2015) |
|  | Spathulenol | 1.1 | Iran | Aerial parts | Esmaeili et. al. (2006) |
|  | Subluteolide | 6.4 - 30.55 | Argentina; Australia; California; Chile; Spain; Turkey | Leaves | Irimia et. al., 2018; Sotes et. al. (2015) |
|  | Taraxasterol | 0.34 - 1.25 | Argentina; Australia; California; Chile; Spain; Turkey | Leaves | Irimia et. al., 2018; Sotes et. al. (2015) |
|  | Taraxasterol acetate | tr - 0.12 | California; Chile; Spain; Turkey | Leaves | Sotes et. al. (2015) |
|  | Terpinene 4-ol | - | Algeria | - | Lograda et. al. (2013) |
|  | Terpineol-4 | <0.05 | Italy | Flower heads | Senatore et. al. (2015) |
|  | *trans*-β-farnesene | - | California | Leaves | Oster et. al. (2015); Beck et. al. (2013) |
|  | Valencene | 0.1 - 1.28 | Algeria; Italy | Flower heads | Senatore et. al. (2015); Lograda et. al. (2013) |
|  | Viridiflorene | <0.05 | Italy | Flower heads | Senatore et. al. (2015) |
|  | α-amyrin | 0.48 - 2.56 | Argentina; Australia; California; Chile; Spain; Turkey | Leaves | Irimia et. al., 2018; Sotes et. al. (2015) |
|  | α-amyrin acetate | tr | California; Chile; Spain; Turkey | Leaves | Sotes et. al. (2015) |
|  | α-cadinol | 1.4 | Iran | Aerial parts | Esmaeili et. al. (2006) |
|  | α-copaene | 0.3 - 2.5 | Algeria; California; Croatia;  Iran;  Turkey | Aerial parts; Flower buds; Flower heads; Leaves | Esmaeili et. al. (2006); Carev et. al. (2017); Buttery et. al. (1986); Binder et. al. (1990); Oster et. al. (2015); Lograda et. al. (2013) |
|  | α-Cubebene | tr | Italy; Turkey | Flower buds; Flower heads | Senatore et. al. (2015); Binder et. al. (1990) |
|  | α-épi muurolol | 0.95 | Algeria | - | Lograda et. al. (2013) |
|  | α-Gurjunene | - | California | Leaves | Oster et. al. (2015); Beck et. al. (2013) |
|  | α-humulene | 0.3 - 0.4 | California;  Iran;  Italy | Aerial parts; Flower heads; Leaves | Esmaeili et. al. (2006); Senatore et. al. (2015); Oster et. al. (2015); Beck et. al. (2013) |
|  | α-muurolene | 0.14 - 0.17 | Turkey | Flower buds; Flower heads | Binder et. al. (1990) |
|  | α-pinene | 0.4 | California; Iran | Aerial parts; Leaves | Esmaeili et. al. (2006); Beck et. al. (2013) |
|  | β- bourbonene | 0.05 | California; Turkey | Flower buds; Leaves | Binder et. al. (1990); Oster et. al. (2015) |
|  | β- Eudesmol | 0.4 | Italy | Flower heads | Senatore et. al. (2015) |
|  | β-amyrin | 0.45 - 2.4 | Argentina; Australia; California; Chile; Spain; Turkey | Leaves | Irimia et. al., 2018; Sotes et. al. (2015) |
|  | β-amyrin acetate | tr - 1.44 | California; Chile; Spain; Turkey | Leaves | Sotes et. al. (2015) |
|  | β-bergamotene *trans* | 0.02 | Algeria | - | Lograda et. al. (2013) |
|  | β-Bisabolene | - | California | Leaves | Oster et. al. (2015) |
|  | β-caryophyllene | 0.43 - 6.2 | Algeria; California; Croatia;  Iran | Aerial parts; Leaves | Esmaeili et. al. (2006); Carev et. al. (2017); Oster et. al. (2015); Beck et. al. (2013); Lograda et. al. (2013) |
|  | β-copaene | 0.03 - 0.04 | California; Turkey | Flower buds; Leaves/stems | Binder et. al. (1990); Beck et. al. (2013) |
|  | β-cubebene | 0.06 - 0.15 | California; Turkey | Flower buds; Flower heads; Leaves/stems | Binder et. al. (1990); Beck et. al. (2013) |
|  | β-damascenone | 0.02 - 0.06 | Turkey | Flower buds; Flower heads; Leaves/stems | Binder et. al. (1990) |
|  | β-Elemen | tr- 0.21 | Algeria; Croatia | Aerial parts | Carev et. al. (2017); Lograda et. al. (2013) |
|  | β-myrcene | - | California | Leaves | Beck et. al. (2013) |
|  | β-pinene | 0.8 | Iran | Aerial parts | Esmaeili et. al. (2006) |
|  | β-Santalene | 0.03 - 0.4 | Italy | Flower buds; Flower heads; Leaves/stems | Senatore et. al. (2015) |
|  | β-selinene | 0.34 - 0.67 | Turkey | Flower buds; Flower heads | Binder et. al. (1990) |
|  | β-sesquifenchene | 0.93 | Algeria | - | Lograda et. al. (2013) |
|  | β-Sesquiphellandrene | - | California | Leaves | Oster et. al. (2015) |
|  | β-Ylangene | 0.6 | Croatia | Aerial parts | Carev et. al. (2017) |
|  | γ-cadinene | 0.08 | California; Turkey | Flower buds; Leaves | Binder et. al. (1990); Beck et. al. (2013) |
|  | γ-Elemene | 0.5 | Croatia | Aerial parts | Carev et. al. (2017) |
|  | γ-Muurolene | 0.3 | California; Italy | Flower heads; Leaves | Senatore et. al. (2015) |
|  | γ-terpinene | 0.56 | Algeria | - | Lograda et. al. (2013) |
|  | δ-Cadinene | 0.03 - 0.48 | California;  Italy;  Turkey | Flower buds; Flower heads; Leaves/stems | Senatore et. al. (2015); Binder et. al. (1990); Beck et. al. (2013) |
|  | Δ-cadinene | 1.78 | Algeria | - | Lograda et. al. (2013) |
|  | δ-Elemene | - | California | Leaves | Beck et. al. (2013) |
| **Nonterpene compounds** | |  |  |  |  |
|  | (E)-1,3-tridecadiene-5,7,9,11-tetrayne | 0.09 - 1.56 | Turkey | Flower buds; Flower heads | Binder et. al. (1990) |
|  | (E)-2-hexenal | 0.05 - 0.27 | California; Turkey | Flower buds; Flower heads; Leaves/stems | Buttery et. al. (1986); Binder et. al. (1990) |
|  | (E)-2-nonenal | 0.04 | Turkey | Flower buds | Binder et. al. (1990) |
|  | (E)-β-ocimene | 0.1 | California | Flower buds | Buttery et. al. (1986) |
|  | (E, E)-2,4-Decadienal | 0.1 | Italy | Flower heads | Senatore et. al. (2015) |
|  | (E,E)-1,3,11-tridecatriene-5,7,9-triyne | 0.1 - 0.57 | Turkey | Flower buds; Flower heads | Binder et. al. (1990) |
|  | (E,E)-1,3,5-tridecatriene-7,9,11-triyne (tentative) | 0.05 - 0.83 | Turkey | Flower buds; Flower heads | Binder et. al. (1990) |
|  | (E,E)-2,4-nonadienal | 0.05 | Turkey | Flower buds | Binder et. al. (1990) |
|  | (E,E,E)-1,3,5,11-tridecatetraene-7,9-diyne | 0.04 - 0.07 | Turkey | Flower buds; Flower heads | Binder et. al. (1990) |
|  | (E,Z,E)-1,3,5,1 1-tridecatetraene-7,9-diyne | 0.22 - 0.23 | Turkey | Flower buds; Flower heads | Binder et. al. (1990) |
|  | (Z)-1,11-tridecadiene-3,5,7,9-tetrayne | 0.02 - 0.04 | Turkey | Flower buds; Flower heads | Binder et. al. (1990) |
|  | (Z)-1,3-tridecadiene-5,7,9,11-tetrayne (tentative) | 0.03 - 0.95 | Turkey | Flower buds; Flower heads | Binder et. al. (1990) |
|  | (Z)-3- hexenol | 0.01 - 0.2 | Turkey | Flower buds; Flower heads; Leaves/stems | Binder et. al. (1990) |
|  | (Z)-3-hexeno | 0.4 | California | Flower buds | Buttery et. al. (1986) |
|  | (Z)-3-hexenyl butanoate | 0.03 | Turkey | Leaves/stems | Binder et. al. (1990) |
|  | (Z)-3-hexenyl propionate | 0.1 | California | Flower buds | Buttery et. al. (1986) |
|  | (Z)-9-Octadecenoic acid | 1.5 | Italy | Flower heads | Senatore et. al. (2015) |
|  | (Z)-β-Damascenone | 0.5 | Italy | Flower heads | Senatore et. al. (2015) |
|  | (Z, Z)-9,12-Octadecadienoic acid | 4.9 | Italy | Flower heads | Senatore et. al. (2015) |
|  | (Z,E)-1,3,11-tridecatrienene-5,7,9-triyne | 0.08 | Turkey | Flower buds; Flower heads | Binder et. al. (1990) |
|  | (Z,E,E)-1,3,5,11-tridecatetraene-7,9-diyne | 0.01 | Turkey | Flower buds | Binder et. al. (1990) |
|  | (Z,Z)-1,8,11-heptadecatriene | 0.29 - 0.77 | Turkey | Flower buds; Flower heads | Binder et. al. (1990) |
|  | (Z,Z,Z)-1,8,11,14-heptadecatetraene | 0.02 - 2.15 | Turkey | Flower buds; Flower heads; Leaves/stems | Binder et. al. (1990) |
|  | 1- heptadecene | 0.04 - 0.09 | Turkey | Flower buds; Flower heads | Binder et. al. (1990) |
|  | 1,1-Bicyclohexyl | 0.08 | Algeria | - | Lograda et. al. (2013) |
|  | 1,2-Benzenedicarboxylic acid, bis(2-methylpropyl) ester | 3.77 | Algeria | - | Lograda et. al. (2013) |
|  | 1,5,9-Trimethyl-1,5,9-cyclododecatriene | - | California | Leaves | Oster et. al. (2015) |
|  | 1-decanol | - | California | Leaves | Beck et. al. (2013) |
|  | 1-pentadecene | 0.2 - 4.9 | California; Turkey | Flower buds; Flower heads; Leaves/stems | Buttery et. al. (1986); Binder et. al. (1990); Oster et. al. (2015) |
|  | 1-tetradecene | 0.06 - 0.23 | Turkey | Flower buds; Flower heads | Binder et. al. (1990) |
|  | 1-tridecene | 0.11 - 0.33 | Turkey | Flower buds; Flower heads | Binder et. al. (1990) |
|  | 1-tridecene-3,5,7,9,11-pentayne (tentative) | 0.33 | Turkey | Flower heads | Binder et. al. (1990) |
|  | 2,4-(E, E)-Octadienal | 0.1 | Italy | Flower heads | Senatore et. al. (2015) |
|  | 2,6,10,14-Hexadecatraen-1-ol,3,7,11,15-tetramethyl-Ac | 0.68 | Algeria | - | Lograda et. al. (2013) |
|  | 2,6,10-Dodecatrien-1-ol,3,7,11-trimethyl- | 1.03 | Algeria | - | Lograda et. al. (2013) |
|  | 2-Cyclohexen-1-one,2-methyl-5-(1-methylethenyl)- | 0.16 | Algeria | - | Lograda et. al. (2013) |
|  | 2-methoxytoluene | 0.2 | California | Flower buds | Buttery et. al. (1986) |
|  | 2-methylbutanoic acid | 0.12 | Turkey | Flower buds | Binder et. al. (1990) |
|  | 2-Methyl-Z,Z-3,13-octadecadienol | 0.71 | Algeria | - | Lograda et. al. (2013) |
|  | 2-pentadecanone-6,10,14-trimethyl | 4.1 | Algeria | - | Lograda et. al. (2013) |
|  | 2-pentanone | 0.04 | Turkey | Flower buds | Binder et. al. (1990) |
|  | 2-Pentyl furan | 0.2 | Italy | Flower heads | Senatore et. al. (2015) |
|  | 2-phenylethanol | 0.04 | Turkey | Flower buds | Binder et. al. (1990) |
|  | 3-methylpentanol | 0.13 | Turkey | Flower buds | Binder et. al. (1990) |
|  | 5-Methylenebicyclo[2,2,1]hept-2-en-7-ylidene acetic acid | 0.39 | Algeria | - | Lograda et. al. (2013) |
|  | 5-β, 8-β,H,9-β, H, 10-α-Labd-14-ene, 8,13-epoxy | 1.16 | Algeria | - | Lograda et. al. (2013) |
|  | 6.9-cis-3,4-epoxy-nonadecadiene (Z,Z) | 3.58 | Algeria | - | Lograda et. al. (2013) |
|  | 6-methylhept-5-en-2-one | 0.07 - 0.19 | Turkey | Flower buds; Flower heads | Binder et. al. (1990) |
|  | 9-(2'2'-Dimethyl propanoilhydrazono)3,6-dichloro-2,7-bis | 0.65 | Algeria | - | Lograda et. al. (2013) |
|  | Aromadendrene oxide | 1.5 | Croatia | Aerial parts | Carev et. al. (2017) |
|  | Benzaldehyde | 0.05 - 0.07 | Turkey | Flower buds; Flower heads; Leaves/stems | Binder et. al. (1990) |
|  | Benzothiazole | 0.03 | Turkey | Leaves/stems | Binder et. al. (1990) |
|  | Calacorene (tentative) | 0.03 | Turkey | Flower buds; Flower heads | Binder et. al. (1990) |
|  | *cis*-3-hexenol | - | California | Leaves | Beck et. al. (2013) |
|  | *cis*-β-ocimene | - | California | Leaves | Beck et. al. (2013) |
|  | Decanal | 0.02 - 1.7 | Croatia;  Italy;  Turkey | Aerial parts; Flower buds; Flower heads; Leaves/stems | Carev et. al. (2017); Senatore et. al. (2015); Binder et. al. (1990) |
|  | Dehydroabietane | 0.66 | Algeria | - | Lograda et. al. (2013) |
|  | Dill apiole | 1.63 | Algeria | - | Lograda et. al. (2013) |
|  | Docosane | tr - 1.39 | Algeria; California; Chile; Spain; Turkey | Leaves | Sotes et. al. (2015); Lograda et. al. (2013) |
|  | Dodecanal | 0.2 | Italy | Flower heads | Senatore et. al. (2015) |
|  | Dotriacontane (branched) | 0.39 - 1.45 | Algeria; Argentina; California; Spain; Turkey | Leaves | Irimia et. al., 2018; Lograda et. al. (2013) |
|  | E-2-heptenal | 0.03 - 0.04 | Turkey | Flower buds; Leaves/stems | Binder et. al. (1990) |
|  | epi-Globulol | <0.05 | Italy | Flower heads | Senatore et. al. (2015) |
|  | Ethylbenzene | 0.05 | Turkey | Flower heads | Binder et. al. (1990) |
|  | Eugenol | 0.3 | Italy | Flower heads | Senatore et. al. (2015) |
|  | Geranyl acetone | 0.8 | Italy | Flower heads | Senatore et. al. (2015) |
|  | Heneicosane | tr | California; Chile; Spain; Turkey | Leaves | Sotes et. al. (2015) |
|  | Hentriacontane (linear or branched) | tr - 3.25 | Argentina; Australia; California; Chile; Croatia; Italy; Spain; Turkey | Aerial parts; Flower buds | Senatore et. al. (2015); Sotes et. al. (2015); Irimia et. al., 2018 |
|  | Heptacosane | 1.34 - 8.1 | Argentina; Australia; California; Chile; Croatia; Italy; Spain; Turkey | Aerial parts; Flower buds | Senatore et. al. (2015) ; Irimia et. al., 2018; Sotes et. al. (2015) |
|  | Heptan-2-one | 0.1 | Italy | Flower heads | Senatore et. al. (2015); Senatore et. al. (2015) |
|  | Hexacosane | tr - 0.8 | California; Chile; Croatia; Italy; Spain; Turkey | Aerial parts; Flower buds | Carev et. al. (2017); Senatore et. al. (2015) ; Sotes et. al. (2015) |
|  | Hexadecanoic acid | 12.79 - 30.8 | Algeria;  Croatia;  Iran;  Italy | Aerial parts; Flower heads | Esmaeili et. al. (2006); Carev et. al. (2017); Senatore et. al. (2015); Lograda et. al. (2013) |
|  | Hexadecanoic acid methyl ester | 3.9 | Italy | Flower heads | Senatore et. al. (2015) |
|  | Hexahydrofarnesyl acetone | 2.8 | Italy | Flower heads | Senatore et. al. (2015) |
|  | Hexanal | 0.19 - 0.48 | Turkey | Flower buds; Leaves/stems | Binder et. al. (1990) |
|  | Hexanol | 0.05 - 0.2 | California; Turkey | Flower buds; Leaves/stems | Buttery et. al. (1986); Binder et. al. (1990); Beck et. al. (2013) |
|  | Lauric acid | 3.08 | Algeria | - | Lograda et. al. (2013) |
|  | Mint sulfide (tentative) | 0.04 - 0.07 | Turkey | Flower buds; Flower heads; Leaves/stems | Binder et. al. (1990) |
|  | Myrestic acid | 3.28 | Algeria | - | Lograda et. al. (2013) |
|  | Myristylaldehyde | 0.72 | Algeria | - | Lograda et. al. (2013) |
|  | Naphtalene | <0.05 | Italy | Flower heads | Senatore et. al. (2015) |
|  | n-heinecosane | 17.3 | Algeria | - | Lograda et. al. (2013) |
|  | Nonacosane (linear or branched) | tr - 8.41 | Argentina; Australia; California; Chile; Croatia; Italy; Spain; Turkey | Aerial parts; Flower buds | Carev et. al. (2017); Senatore et. al. (2015); Sotes et. al. (2015); Irimia et. al., 2018 |
|  | Nonanal | 0.05 - 1.4 | Croatia;  Italy;  Turkey | Aerial parts; Flower buds; Flower heads; Leaves/stems | Carev et. al. (2017); Senatore et. al. (2015); Binder et. al. (1990) |
|  | n-pentacosane | 5.64 | Algeria | - | Lograda et. al. (2013) |
|  | n-tricosane | 10.51 | Algeria | - | Lograda et. al. (2013) |
|  | Oct-1-en-3-ol | 0.12 | Turkey | Flower buds | Binder et. al. (1990) |
|  | Octacosane | 0.06 - 0.9 | California; Chile; Croatia; Italy; Spain; Turkey | Aerial parts; Flower buds | Carev et. al. (2017); Senatore et. al. (2015) ; Sotes |
|  | Octadecanoic acid | 0.6 | Italy | Flower heads | Senatore et. al. (2015) |
|  | Octan-3-ol | <0.05 | Italy | Flower heads | Senatore et. al. (2015) |
|  | Octanal | tr - 0.17 | Italy; Turkey | Flower buds; Flower heads; Leaves/stems | Senatore et. al. (2015) ; Binder et. al. (1990) |
|  | Octane,2-methyl- | 0.6 | Algeria | - | Lograda et. al. (2013) |
|  | Pentacosane | 0.11 - 5.2 | California; Chile; Croatia; Italy; Spain; Turkey | Aerial parts; Flower buds | Carev et. al. (2017); Senatore et. al. (2015) ; Irimia et. al., 2018; Sotes et. al. (2015) |
|  | Pentadecanal | 0.9 | Italy | Flower heads | Senatore et. al. (2015) |
|  | Pentadecane | 1.7 | Italy | Flower heads | Senatore et. al. (2015) |
|  | Pentadecanoic acid | 0.8 | Italy | Flower heads | Senatore et. al. (2015) |
|  | Perillene (tentative) | 1 | California | Flower buds | Buttery et. al. (1986) |
|  | Phenyl acetaldehyde | 0.05 - 1.8 | Italy; Turkey | Flower buds; Flower heads; Leaves/stems | Senatore et. al. (2015) ; Binder et. al. (1990) |
|  | Phytol | 3.5 | Croatia | Aerial parts | Carev et. al. (2017) |
|  | *p*-Vinyl guaiacol | 0.3 | Croatia | Aerial parts | Carev et. al. (2017) |
|  | Tetracosane | tr - 0.6 | California; Chile; Croatia; Italy; Spain; Turkey | Aerial parts; Flower buds | Carev et. al. (2017); Senatore et. al. (2015) ; Sotes et. al. (2015) |
|  | Tetradecanal | 1.3 - 2.1 | Croatia; Italy | Aerial parts; Flower heads | Carev et. al. (2017); Senatore et. al. (2015) |
|  | Tetradecane | 0.8 | Italy | Flower heads | Senatore et. al. (2015) |
|  | Tetradecanoic acid | 2.5 | Italy | Flower heads | Senatore et. al. (2015) |
|  | Tetradecanol | 1.3 | Italy | Flower heads | Senatore et. al. (2015) |
|  | *trans*-2-hexenal | - | California | Leaves | Beck et. al. (2013) |
|  | *trans*-2-hexenol | - | California | Leaves | Beck et. al. (2013) |
|  | *trans*-β-ocimene | - | California | Leaves | Beck et. al. (2013) |
|  | Triacontane | tr - 0.18 | California; Chile; Spain; Turkey | Leaves | Sotes et. al. (2015) |
|  | Tricosane | tr - 2.9 | California; Chile; Croatia; Italy; Spain; Turkey | Aerial parts; Flower buds | Carev et. al. (2017); Senatore et. al. (2015) ; Sotes et. al. (2015) |
|  | Tridecane | 0.6 | Italy | Flower heads | Senatore et. al. (2015) |
|  | Tritriacontane (branched) | 0.6 - 1.67 | Argentina; Australia; California; Spain; Turkey | Leaves | Irimia et. al., 2018 |
|  | α-Linolenic acid | 17.9 | Croatia | Aerial parts | Carev et. al. (2017) |
|  | α-tridecene | 0.54 | Algeria | - | Lograda et. al. (2013) |
